# Upper gastrointestinal triple stenosis in a patient with trisomy 17p syndrome: Case report and literature review

**DOI:** 10.1002/deo2.70043

**Published:** 2024-12-23

**Authors:** Hiroko Ando, Hideki Mori, Kaoru Takabayashi, Noriko Matsuura, Tatsuhiro Masaoka, Juntaro Matsuzaki, Yoshimasa Saito, Motohiko Kato, Kenjiro Kosaki, Takanori Kanai

**Affiliations:** ^1^ Department of Internal Medicine Division of Gastroenterology and Hepatology Keio University School of Medicine Tokyo Japan; ^2^ Center for Diagnostic and Therapeutic Endoscopy Keio University School of Medicine Tokyo Japan; ^3^ Division of Research and Development for Minimally Invasive Treatment Cancer Center Keio University School of Medicine Tokyo Japan; ^4^ Center for Medical Genetics Keio University School of Medicine Tokyo Japan

**Keywords:** duodenal web, gastric ulcer, gastroesophageal reflux disease, trisomy 17p syndrome, upper gastrointestinal stenosis

## Abstract

Upper gastrointestinal stenosis, which can be congenital or acquired, can lead to dysphagia. The association between trisomy 17p syndrome, a rare chromosomal abnormality, and upper gastrointestinal stenosis is unclear. A 20‐year‐old man diagnosed with trisomy 17p syndrome was referred to our department due to recurrent vomiting. Esophagogastroduodenoscopy revealed stenotic areas in the esophagus, stomach, and duodenum. The congenital gastrointestinal stenosis present in both the duodenum and esophagus suggested that the stasis and reflux of digestive fluids exacerbated stenosis in the stomach and esophagus. Gastric acid suppression therapy and endoscopic dilation of the esophagus and duodenum effectively resolved the patient's vomiting symptoms.

## INTRODUCTION

Upper gastrointestinal stenosis may be congenital or acquired and, when severe, can cause dysphagia. Trisomy 17p syndrome is a rare chromosomal abnormality,^1^ but its association with upper gastrointestinal stenosis is not clear. This report describes a rare case of upper gastrointestinal triple stenosis in a patient with trisomy 17p syndrome.

### Case report

A 20‐year‐old man with a duplication of the short arm of chromosome 17 was referred to our center because of repeated vomiting. The patient had presented since childhood with mental retardation, hypotonia, ptosis, microgenia, hypospadias, and scrotal hydrocele associated with trisomy 17p syndrome. At age 10, the patient underwent surgery for hypospadias. The patient had no history of steroid or nonsteroidal anti‐inflammatory drug use. The patient had experienced vomiting symptoms since around age 15 and had been hospitalized at another hospital approximately once a year for treatment of these symptoms. The patient was initially diagnosed with superior mesenteric artery syndrome based on X‐ray findings.

**FIGURE 1:**
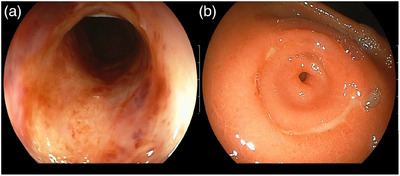
Upper gastrointestinal endoscopic findings in this patient 4 years prior to the current presentation, showing. Circumferential inflammation with accompanying ulcers in the thoracic esophagus. A ring‐shaped ulcer in the antrum surrounding the pylorus.

An upper gastrointestinal endoscopy performed 4 years earlier, at age 16 years, to investigate hematemesis revealed erosions and ulcers in the middle thoracic esophagus and a ring ulcer in the pyloric antrum (Figure 1a,b). Further examination distal to the pylorus was not conducted at that time. The patient was subsequently treated with H2 blockers, and although hematemesis did not recur, episodes of vomiting continued to occur periodically.

**FIGURE 2:**
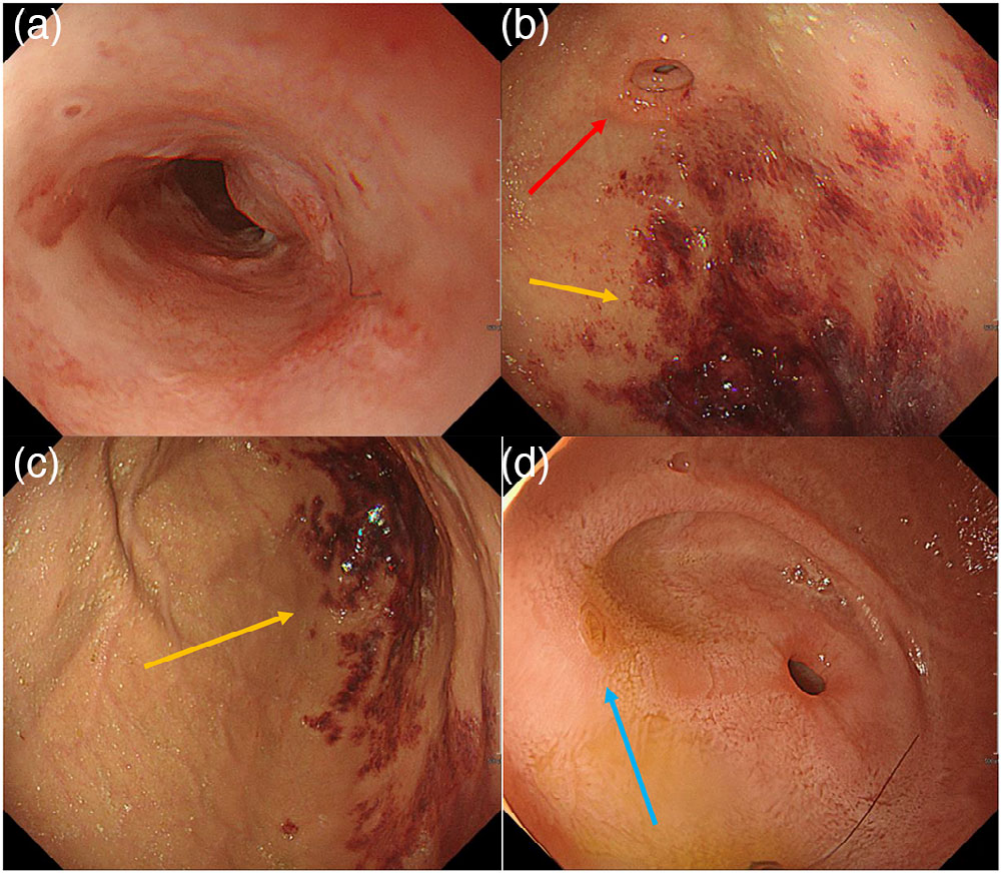
Initial upper gastrointestinal endoscopic findings in this patient at the time of initial presentation, showing. (a) Stenosis is associated with ulcers in the thoracic esophagus, with the mucosal epithelium exhibiting circumferential inflammatory changes. (b, c) Ischemic changes and pseudo‐pylorus in the gastric antrum. The red arrow indicates the pseudo‐pylorus, and the orange arrow indicates ischemic changes. (d) Membrane‐like stenosis in the descending part of the duodenum. The membrane‐like stenosis was located near the distal side of the duodenal papilla. The blue arrow indicates the duodenal papilla.

**FIGURE 3:**
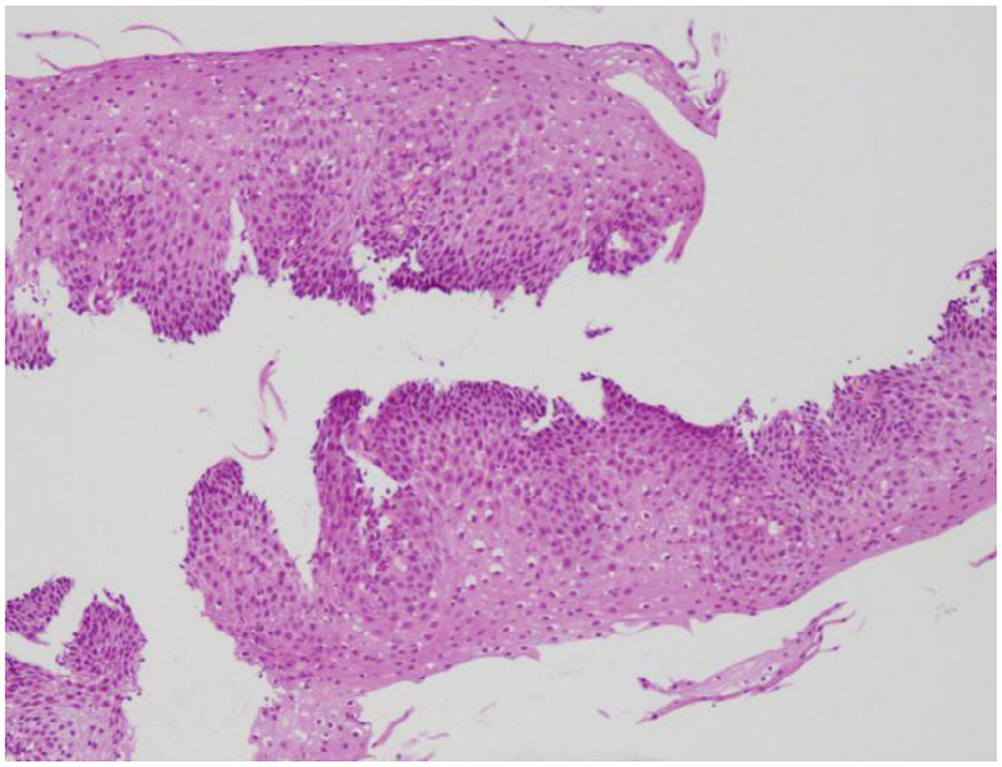
Histopathological findings at the margins of the esophageal ulcer. Hematoxylin and eosin staining showed enlargement of nuclei in the epithelial cells of the basal layer and homogeneous morphology and distribution of these cells.

An upper gastrointestinal endoscopy was conducted at age 20 due to episodes of repeated vomiting, black vomitus, and anemia, revealing an esophageal ulcer and stenosis in the middle thoracic esophagus (Figure 2a), ischemic changes, and a pseudo‐pylorus in the gastric antrum (Figure 2b,c), and duodenal membrane‐like stenosis (Figure 2d). Although the lower esophagus appeared slightly edematous, no erosions or ulcers were observed. He was negative for *Heliobacter pylori* infection by the gastric juice polymerase chain reaction method. Histological evaluation of the esophageal ulcer margins revealed only inflammatory cell infiltration and reactive changes, with no evidence of eosinophilic infiltration or malignancy (Figure 3). The duodenal membrane‐like stenosis was impassable even with a small‐diameter endoscope (GIF 1200N, distal‐end outer diameter 5.4 mm; Olympus Medical System). Computed tomography showed the absence of an annular pancreas. The patient was treated with a proton pump inhibitor in place of an H2 blocker, enabling him to again eat food, and he was discharged from the hospital.

**FIGURE 4:**
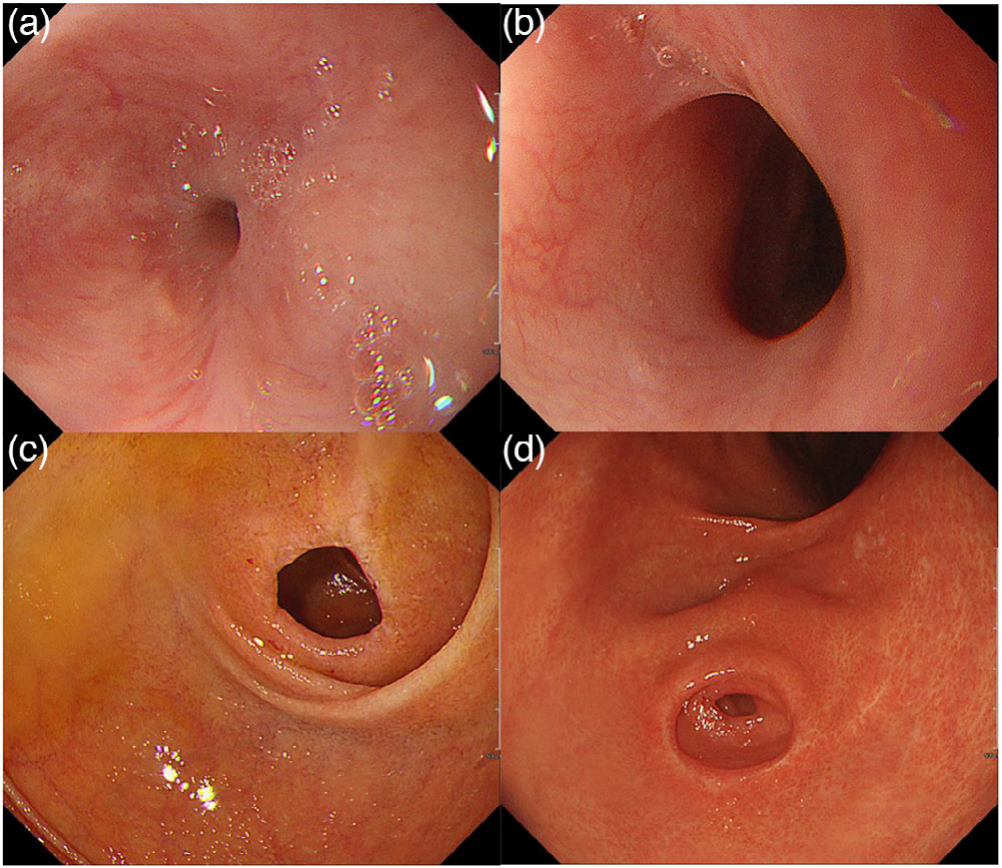
Upper gastrointestinal endoscopic findings in this patient after treatment, showing. (a) Severe scar‐induced stenosis in the thoracic esophagus 1 month after conservative treatment with acid secretion inhibitors. (b) Adequate esophageal expansion, following repeat balloon dilation of esophageal stenosis. (c) Dilation of the membrane‐like stenosis of the duodenum using a scope bougie. (d) Recovery of ischemic changes, with no changes in the pseudo‐pylorus of the stomach.

One month later, the patient was again referred to our hospital for frequent vomiting. An upper gastrointestinal endoscopy showed severe scar stenosis in the thoracic esophagus, rendering the passage of a small‐diameter endoscope impossible (GIF 1200N, distal‐end outer diameter 5.4 mm; Olympus Medical System; Figure 4a). The esophageal scar stenosis was attributed to the healing of esophageal ulcers and erosions. The chronic inflammation of the esophagus observed in this patient was thought to be caused by gastroesophageal reflux resulting from the passage obstruction associated with duodenal membrane‐like stenosis. The duodenal membrane‐like stenosis in this patient was dilated with a scope bougie (GIF H‐290, distal‐end outer diameter 8.9 mm; Olympus Medical System), whereas his esophageal stenosis was treated by balloon dilation (Figure 4b,c). Since then, the patient has been able to ingest food, but his esophageal stenosis has required regular repeated balloon dilation. Duodenal scope bougienage achieved satisfactory dilation in a single session; however, esophageal balloon dilation was performed a total of 10 times at intervals of 1–2 weeks. The pseudo‐pylorus in the gastric antrum did not change during the course of treatment, suggesting that the pseudo‐pylorus was chronic. Over time, the ischemic changes surrounding the pseudo‐pylorus have healed (Figure 4d).

## DISCUSSION

Trisomy 17p syndrome is a rare genetic disorder marked by physical and developmental abnormalities, such as growth and motor delays, skeletal anomalies, and distinctive facial features. Due to its extreme rarity, data on its incidence, life expectancy, and survival rates are lacking; however, most reported cases result in early childhood mortality, suggesting a poor prognosis.^1^, ^2^ Less is known, however, about the associations between this syndrome and gastrointestinal disorders, particularly stenotic lesions and ulcerative diseases. The patient described in this report was found to have stenotic lesions in three different upper gastrointestinal organs: the esophagus, stomach, and duodenum.

Evaluation of the relationships between esophageal stenosis and ulcerative lesions showed that inflammation was most intense in the mid‐esophagus and less severe at the esophagogastric junction, characteristics differing from those typically observed in patients with reflux esophagitis.

Esophageal atresia/stenosis occurs in one in 25,000–50,000 births as a congenital disorder. Trisomy 21 is found in 11.1% of these patients.^4^ Congenital esophageal stenosis can be divided into three pathohistological types: tracheobronchial remnants (TBR); fibromuscular thickening or fibromuscular stenosis (FMS); and membranous webbing or esophageal membrane.^5^ The endoscopic findings in this patient suggest the likelihood of TBR or FMS, although both require pathological evaluation of deeper layers beyond the submucosa. Although a definitive diagnosis could not be established in this patient, his clinical course and histopathological findings suggest that drug‐induced esophagitis, eosinophilic esophagitis, and malignant tumors were unlikely causes of his esophageal stenosis.

The differential diagnosis of esophageal ulcers includes reflux esophagitis, drug‐induced ulcers, infectious ulcers, malignant tumors, Behçet's disease, and Crohn's disease.^6^ The findings in this patient, of duodenal or gastric stenotic lesions leading to chronic upper gastrointestinal passage disorders, suggest the likelihood of reflux esophagitis exacerbating the stenotic area associated with congenital esophageal stenosis. To date, however, there have been no reports on the potential fragility of the mucosa in trisomy 17p syndrome, necessitating the evaluation of additional patients.

The formation of gastric pseudo‐pylorus is thought to result from repeated inflammation, including ulcers at the same site. Indeed, a ring ulcer had been previously observed in this area of the present patient. It is known that bile and pancreatic reflux increase the risk of worsening gastric ulcers.^9^, ^10^ In this case, the presence of duodenal stenosis and frequent episodes of vomiting suggest a strong possibility that chronic reflux of bile and pancreatic juices contributed to the development of chronic gastric ulcers. Other possible causes of chronic gastric ulcers include H. pylori infection, drug‐induced gastric ulcers, or bacterial gastroenteritis. However, H. pylori infection and drug‐induced gastric ulcers are unlikely based on the patient's medical history and endoscopic findings, and repeated bacterial gastroenteritis limited to the antrum is also improbable.

The morphology and progression of membranous stenosis in this patient suggest that his duodenal stenosis was likely congenital.^9^ Congenital duodenal atresia/stenosis has been estimated to occur in one of 9000–40,000 persons.^4^ Congenital duodenal obstruction is a common type of neonatal intestinal obstruction, primarily caused by intrinsic duodenal atresia and stenosis. Duodenal obstruction may also be caused by extrinsic factors, including peritoneal bands. Other causes include duodenal diaphragm, malrotation, and annular pancreas. Congenital duodenal obstruction is frequently associated with other congenital anomalies, particularly cardiac defects and Down syndrome.^10^

Vomiting symptoms in the present patient were effectively managed through a combination of scope bougie dilation of the membranous stenosis of the duodenum, multiple balloon dilations of the scar‐induced esophageal stenosis, and oral administration of acid secretion inhibitors. As a result, vomiting symptoms did not recur and the patient's dietary intake improved significantly. Other options for esophagus stenosis include endoscopic stent placement, esophagectomy, and esophageal reconstruction. For the duodenal stenotic areas, a gastrojejunostomy may be considered. Endoscopic stent placement offers advantages such as being less invasive than surgery and providing immediate relief. However, it has drawbacks, including the risk of restenosis, discomfort or pain, infection, and bleeding, particularly in benign stenoses, issues such as stent migration and poor fixation. Surgical treatment is effective in resolving stenoses, but it carries high invasiveness and risks of complications like anastomotic leakage, anastomotic restenosis, and ulcer formation. Selecting an appropriate treatment approach requires careful consideration of the pathology and severity of the condition.

For patients with trisomy 17p syndrome, an effective approach to preventing or detecting gastrointestinal stenosis early should involve identifying early symptoms, such as changes in food intake or vomiting. Relatively non‐invasive options, such as gastrointestinal contrast studies or computed tomography scans, may be suitable for screening and identifying stenotic sites. However, as demonstrated in this case, where the condition was initially misdiagnosed as superior mesenteric artery syndrome, early consideration of endoscopy is warranted if stenosis is suspected. Thus, the accumulation of additional data on gastrointestinal lesions in this syndrome is anticipated.

## CONFLICT OF INTEREST STATEMENT

None.

## ETHICS STATEMENT

All procedures were performed in accordance with the ethical standards of the Declaration of Helsinki and its later amendments.

## PATIENT CONSENT STATEMENT

Informed consent was obtained from the patient for the publication of this case report.
